# A male rat model integrating hypertension, chronic cerebral hypoperfusion, and aging recapitulates multimodal cerebral small vessel disease phenotypes

**DOI:** 10.3389/fnagi.2026.1906043

**Published:** 2026-08-14

**Authors:** Zhiwei Feng, Shan Han, Yanyi Li, Zhenzhen Li, Yige Tu, Bin Wu, Shengjie Zhu, Qin Zheng, Wenjun Sun

**Affiliations:** 1 Beijing University of Chinese Medicine, Beijing, China; 2 Beijing Miyun District Hospital of Traditional Chinese Medicine, Beijing, China; 3 Department of Encephalopathy, Beijing University of Chinese Medicine Third Affiliated Hospital, Beijing, China

**Keywords:** animal model, cerebral small vessel disease, chronic cerebral hypoperfusion, D-galactose, hypertension, white matter injury

## Abstract

**Background:**

Cerebral small vessel disease (CSVD) usually develops under the combined influence of vascular risk factors, chronic perfusion insufficiency, and age-related brain vulnerability; however, animal models driven by a single insult do not fully reproduce this multifactorial clinical context.

**Methods:**

We established a composite CSVD rat model by combining spontaneous hypertension, bilateral common carotid artery stenosis (BCAS)-induced chronic cerebral hypoperfusion, and D-galactose (D-gal)-induced aging-related stress. Fourteen-week-old male Wistar-Kyoto (WKY) rats and spontaneously hypertensive rats (SHRs) were assigned to the WKY, SHR, SHR+BCAS, and SHR+BCAS+D-gal groups. Spatial learning and memory, exploratory behavior, spontaneous locomotion, and motor coordination were assessed using the Morris water maze, open field test, and balance beam test. T2-weighted magnetic resonance imaging (MRI) and diffusion tensor imaging (DTI) were used to quantify regional brain volumes and white matter microstructural integrity. Luxol fast blue (LFB) staining and transmission electron microscopy (TEM) were performed to examine corpus callosum myelin pathology, and enzyme-linked immunosorbent assay (ELISA) was used to assess molecular markers related to the blood-brain barrier (BBB), neuroinflammation, oxidative stress, myelin injury, axonal injury, and neuronal damage.

**Results:**

Compared with WKY rats, SHR rats showed early spatial memory impairment, behavioral abnormalities, and structural brain changes. Superimposition of BCAS and D-gal progressively aggravated these abnormalities. SHR+BCAS+D-gal rats exhibited the longest escape latency, reduced target-quadrant dwell time and platform crossings, decreased locomotor and exploratory activity, prolonged balance beam crossing time, increased foot slips, and greater waiting/stopping behavior. MRI revealed ventricular enlargement and reduced hippocampal, cortical, and corpus callosum volumes. DTI showed lower fractional anisotropy in the genu of the corpus callosum, cingulum, dorsal hippocampus, and thalamic core region. LFB staining showed reduced myelin staining and disorganized fiber arrangement, whereas TEM revealed myelin lamellar loosening, irregularity, and deformation in the corpus callosum. Molecular analyses showed decreased Claudin-5, ZO-1, MBP, and SOD, and increased Iba1, GFAP, MDA, corpus callosum NfL, serum NfL, and serum NSE, especially in the SHR+BCAS+D-gal group.

**Conclusion:**

In male rats, the SHR+BCAS+D-gal composite model recapitulates the multifactorial background, chronic progression, and multimodal neurological impairment of clinical CSVD at behavioral, neuroimaging, histopathological, and molecular levels. This male rat model may provide a useful experimental platform for mechanistic and interventional studies targeting BBB protection, neuroinflammation, oxidative stress, and white matter repair.

## Introduction

Cerebral small vessel disease (CSVD) is a common cerebrovascular disorder in older adults that primarily affects small arteries, arterioles, capillaries, and small veins in the brain. Its typical neuroimaging manifestations include white matter hyperintensities, lacunes, cerebral microbleeds, enlarged perivascular spaces, and brain atrophy (Duering et al., 2023). CSVD is not merely an incidental imaging abnormality; it is an important pathological substrate for lacunar stroke, intracerebral hemorrhage, vascular cognitive impairment, and dementia (Elahi et al., 2023). In addition to cognitive decline, patients with CSVD often present with multidimensional neurological dysfunction, including depression, apathy, bradykinesia, gait disturbance, impaired balance, increased fall risk, and reduced activities of daily living (Kancheva et al., 2024). Recent clinical neuroimaging evidence further suggests that cognitive impairment, apathy, and gait disturbance in CSVD may not be independent phenomena, but may instead reflect shared injury to frontal-subcortical circuits and white matter network connectivity (Li et al., 2024). Therefore, a clinically relevant animal model of CSVD should reproduce not only vascular lesions or white matter injury, but also the multidimensional behavioral and imaging phenotypes observed in patients.

The development and progression of CSVD are usually not driven by a single pathological factor, but by the long-term interaction of vascular risk factors, abnormal cerebral perfusion, and age-related degenerative changes. Hypertension and aging are widely recognized as the two most important risk factors for sporadic CSVD. They promote arteriolosclerosis, vascular remodeling, endothelial dysfunction, increased BBB permeability, and impaired regulation of the cerebral microcirculation, eventually leading to white matter injury, brain atrophy, and cognitive decline (Hainsworth et al., 2024). Moreover, chronic cerebral hypoperfusion can induce sustained cerebral ischemia and hypoxia, energy metabolism disturbance, and oxidative stress, thereby further triggering BBB disruption, glial activation, myelin damage, and impaired axonal integrity; these processes represent major pathological mechanisms underlying vascular cognitive impairment and CSVD-like white matter lesions (Rajeev et al., 2023). In addition, aging itself reduces cerebrovascular reactivity and neurovascular unit homeostasis, and increases the vulnerability of white matter and neurons to hypoxic-ischemic injury through oxidative stress, inflammation, and mitochondrial dysfunction (Shwe et al., 2018). Thus, from a disease-mechanism perspective, clinical CSVD is better understood as a chronic progressive cerebrovascular-neurodegenerative disorder jointly driven by hypertension, hypoperfusion, and aging, rather than as a focal brain injury caused by a single insult.

Current experimental models related to CSVD mainly include hypertension-associated models, chronic cerebral hypoperfusion models, hereditary small-vessel disease models, and aging-related models. These models have provided important platforms for elucidating different pathological components of CSVD and vascular cognitive impairment (Hainsworth and Markus, 2008; Hainsworth et al., 2017). Among hypertension-associated models, stroke-prone spontaneously hypertensive rats (SHRSP) have been widely used to model severe hypertensive small-vessel pathology and spontaneous stroke-related phenotypes, but this strain may overrepresent the severe ischemic or hemorrhagic aspects of CSVD. By contrast, spontaneously hypertensive rats (SHRs), the parental strain of SHRSP, develop chronic hypertension and relatively mild early CSVD-like brain changes, including white matter injury, cognitive impairment, and neuroinflammatory alterations (Kaiser et al., 2014). Importantly, SHR should not be viewed as a single-risk-factor model in a strict sense, because this strain may also exhibit additional cardiovascular and metabolic abnormalities, including altered lipid metabolism and age-related atrial arrhythmogenic remodeling (Iritani et al., 1977; Lau et al., 2013). In the present study, SHR was therefore used as a defined hypertensive and cardiovascular-risk background rather than as a purely isolated hypertension model. BCAS induces relatively stable chronic cerebral hypoperfusion and causes white matter injury and spatial working memory impairment, making it an important model for vascular cognitive impairment and ischemic white matter disease (Shibata et al., 2007). D-galactose (D-gal)-induced aging models can mimic aging-related oxidative stress, neuroinflammation, and cognitive decline, and are widely used to investigate brain aging and age-related neurodegenerative changes (Cai et al., 2022). Thus, SHR, BCAS, and D-gal each emphasize different components of the CSVD pathological cascade, including cardiovascular-risk background, chronic cerebral hypoperfusion, and aging-related vulnerability. However, none of these approaches alone fully recapitulates the combined influence of multiple vascular risk factors, chronic perfusion insufficiency, aging-related brain vulnerability, white matter injury, structural brain changes, cognitive decline, behavioral abnormalities, and gait-balance dysfunction commonly seen in patients with CSVD.

Against this background, the present study aimed to establish a composite CSVD rat model by combining BCAS and D-gal-induced aging-related stress on the basis of spontaneous hypertension. This design was not intended as a simple accumulation of modeling procedures, but rather as an attempt to reproduce the pathological environment in which chronic hypertension, insufficient cerebral microcirculatory perfusion, and age-related brain vulnerability coexist in patients with CSVD. We hypothesized that, on a background of hypertension-induced structural and functional vulnerability of cerebral small vessels, chronic cerebral hypoperfusion would further aggravate brain ischemia, hypoxia, and energy metabolic disturbance, whereas aging-related stress would amplify oxidative stress, inflammatory responses, and disruption of neurovascular unit homeostasis. The convergence of these three factors may generate a continuous pathological chain involving vascular injury, BBB disruption, neuroinflammation and oxidative stress, white matter myelin and axonal injury, and cognitive, emotional, and motor dysfunction. Accordingly, the SHR+BCAS+D-gal composite model may more comprehensively mimic the chronic progression and multidimensional injury profile of clinical CSVD than single-risk-factor models.

Therefore, this study performed a multimodal phenotypic characterization of the SHR+BCAS+D-gal composite-risk-factor CSVD rat model. Behavioral tests, 7.0-T MRI/DTI, histopathology, and molecular assays were used to evaluate cognitive function, emotional/exploratory behavior, motor balance, structural brain changes, white matter microstructural injury, and myelin/axonal damage. The aim was to clarify the experimental value of this model for simulating multidimensional neurological dysfunction and white matter pathology in clinical CSVD. Notably, this study extends prior work by providing a systematic multimodal characterization encompassing 7.0-T DTI fractional anisotropy mapping, TEM ultrastructural analysis of myelin lamellae, serum neurofilament light chain and NSE quantification, and a dedicated balance beam protocol – dimensions that have not been simultaneously addressed in previous composite CSVD models.

## Materials and methods

### Animals

Fourteen-week-old adult male SHRs (*n* = 36) and age-matched normotensive Wistar-Kyoto (WKY) rats (*n* = 12), weighing 280–320 g, were purchased from Beijing Vital River Laboratory Animal Technology Co., Ltd. All rats were housed in a standard specific pathogen-free animal facility under controlled temperature (20–22 °C) and humidity (50 ± 10%), with a 12-h light/dark cycle and free access to food and water. All animal experimental procedures were approved by the Experimental Animal Ethics Subcommittee of the Academic Committee of Beijing University of Chinese Medicine (approval no. BUCM-2025111901-4024) and were performed in accordance with institutional animal welfare and ethics guidelines.

### Model establishment

Chronic cerebral ischemia was induced using a modified BCAS procedure (Zhilan et al., 2024). The efficacy of CCA stenosis was confirmed by surgical verification of ligation tightness (Matin et al., 2016). Direct cerebral blood flow measurement was not performed in this study. Rats were anesthetized by mask inhalation of 3% isoflurane in medical oxygen and fixed in the supine position. A midline cervical incision was made, and the bilateral common carotid arteries (CCAs) were exposed and bluntly separated. A miniature steel wire with an outer diameter of 0.5 mm was placed parallel to the left CCA, and the vessel and wire were tightly ligated together using 4-0 silk sutures. The wire was then gently withdrawn to induce arterial stenosis. The same procedure was repeated on the right CCA. All operations were performed on a feedback-controlled heating pad to maintain body temperature at approximately 37 °C, and each procedure was completed within 20 min. Rats in the WKY and SHR groups underwent exposure and isolation of the vessels without ligation.

To incorporate aging-related stress, rats in the SHR+BCAS+D-gal group received subcutaneous D-galactose (D-gal; Solarbio, Beijing, China) at 400 mg/kg/day starting 1 week after surgery for 7 consecutive weeks. This dose was selected based on previous studies demonstrating that subcutaneous D-gal at 400 mg/kg/day reliably induces oxidative stress, neuroinflammation, and cognitive decline in rodents without causing overt systemic toxicity (Yuan et al., 2020). Rats in the other groups received an equal volume of normal saline. All subsequent experiments and data analyses were performed under blinded conditions.

### Experimental design, randomization, blinding, and sample size

A total of 48 rats were included in this study, comprising 12 WKY rats and 36 SHRs. After 1 week of acclimatization, SHRs were assigned by a random number table to the SHR, SHR+BCAS, and SHR+BCAS+D-gal groups (*n* = 12 per group), while WKY rats served as the normotensive control group. Sample sizes were determined based on preliminary experiments and previous studies of similar models, while allowing for BCAS-related mortality, modeling failure, and tissue loss during downstream assays.

Behavioral tests were performed in a pre-specified balanced cohort of 10 rats per group. In the WKY and SHR groups, all 12 animals survived; however, 10 animals per group were included in the behavioral cohort to match the number of surviving animals available in the BCAS-containing groups and to maintain balanced group sizes for behavioral comparisons. The remaining two animals in the WKY and SHR groups were not excluded because of health status, behavioral performance, missing data, or data-quality concerns.

For MRI and post-mortem investigations, animals were selected from the eligible animals in each group using a random-number table before outcome analysis. Six animals per group underwent MRI and ELISA-based molecular assays, and three animals per group were used for LFB staining and TEM observation. These sample sizes were pre-specified according to feasibility, cost, tissue-processing requirements, and the exploratory multimodal nature of the study. Animals not selected for a particular downstream assay were not excluded on the basis of behavioral performance, model severity, imaging findings, tissue quality, or other outcome-related criteria.

Behavioral testing, MRI image analysis, pathological image acquisition and quantification, TEM observation, and ELISA data processing were conducted by investigators blinded to group allocation. Inclusion criteria were good general condition, body weight within the experimental range, and absence of obvious infection, trauma, or neurological abnormality. Exclusion criteria included intraoperative or postoperative death, severe infection, persistent body weight loss, inability to eat or drink independently, poor-quality MRI images, missing behavioral data, or tissue samples unsuitable for analysis. All deaths, exclusions, and sample losses were recorded and reported in the Results section.

### Blood pressure measurement

Systolic and diastolic blood pressure were measured indirectly using a non-invasive tail-cuff blood pressure system at weeks 0, 2, 4, 6, and 8. During measurement, rats were placed in a warming device for restraint. The tail skin was wiped with 75% ethanol to remove thick keratinized tissue, and the sensor and pulse transducer were then fixed around the tail. After the rats became quiet, blood flow signals were recorded to obtain blood pressure values. Measurements were performed between 8:00 and 17:00. Each rat was measured five consecutive times, and the mean of the remaining values after removal of outliers was used as the final blood pressure value.

### Morris water maze

The Morris water maze (MWM) was used to assess spatial learning and memory. The apparatus consisted of a circular pool (1.8 m in diameter and 0.6 m in height), an image acquisition system, and VisuTrack analysis software (Shanghai Xinruan, China). The pool was filled with blackened water maintained at 22–25 °C. A hidden circular platform (10 cm in diameter) was placed 0.5 cm below the water surface. The place navigation test was conducted for 5 consecutive days (days 1–5). Rats were released into the water facing the wall of the pool, and the time required to locate the hidden platform was recorded as the escape latency. Four trials were performed each day, with the starting position changed for each trial. If a rat located the platform within 60 s, it was allowed to remain on the platform for an additional 30 s. If it failed to locate the platform within 60 s, it was guided to the platform by the experimenter and allowed to remain there for 30 s; the escape latency for that trial was recorded as 60 s. On day 6, the platform was removed for the probe trial. Rats were released from the same quadrant, and target-quadrant dwell time and the number of crossings over the former platform location were recorded as indices of spatial memory retention.

### Open field test

The open field test was used to evaluate spontaneous locomotion, exploratory behavior, and anxiety-related behavior. The apparatus was a black, open-top box measuring 100 cm × 100 cm × 40 cm. The test was conducted in a quiet room. The floor of the arena was virtually divided by VisuTrack video-tracking software into 16 equal squares (25 cm × 25 cm), with the central four squares defined as the central zone and the remaining 12 squares as the peripheral zone. Each rat was gently placed at the geometric center of the arena, after which the experimenter immediately left the field of view. The software automatically tracked and recorded behavior for 5 min, including total distance traveled, time spent in the central zone, number of central-zone entries, and number of rearings. After each test, feces and urine were removed, and the box was thoroughly wiped with 75% ethanol to eliminate residual odors.

### Balance beam test

The balance beam test was used to assess fine motor coordination, body balance, and hesitation during locomotion. The device was suspended 1 m above the floor and consisted of an 80-cm-long horizontal beam connected at the end to a rectangular dark box containing standard chow as an incentive. A bright light was placed above the starting point as an aversive stimulus to encourage rats to move toward the dark box. The test lasted 3 days, with the first 2 days used for adaptation and training and the third day for formal testing. During formal testing, each rat was placed at the starting point of the beam, and the time from leaving the starting point to complete entry of all four limbs into the dark box was recorded as the crossing time. If the rat failed to reach the dark box within 60 s, the crossing time was recorded as 60 s. The number of hindlimb slips or falls from the beam was recorded as the number of foot slips. To further characterize motor execution and hesitation, active movement time and waiting/stopping time were also measured. Active movement time was defined as the cumulative duration of active forward movement on the beam, including continuous walking, slow progression, and continued forward movement after gait adjustment. Waiting/stopping time was defined as the cumulative duration of immobility, hesitation, pauses, or failure to move forward at the starting point or during beam crossing. All behaviors were recorded by a video acquisition system and analyzed offline by investigators blinded to group allocation. Each rat completed three formal trials with an inter-trial interval of 1 min, and the average value was used for statistical analysis.

### MRI acquisition and analysis

Brain imaging was performed using a 7.0-T small-animal MRI scanner. During MRI acquisition, rats were anesthetized with isoflurane delivered in medical oxygen using a nose cone. Anesthesia was induced with 3.0% isoflurane and maintained with 1.5–2.0% isoflurane during scanning. Rats were securely fixed in the prone position on the scanning bed. Body temperature was maintained at approximately 37 °C using a heating system, and respiration was monitored throughout the procedure, with the depth of anesthesia adjusted as needed to maintain stable breathing.

T2-weighted structural imaging: High-resolution T2-weighted images (T2WI) were acquired using a spin-echo sequence. The scanning parameters were as follows: repetition time (TR) = 4,200 ms, echo time (TE) = 36 ms, field of view (FOV) = 3.5 cm × 3.5 cm, matrix = 256 × 256, slice thickness = 0.8 mm, inter-slice gap = 1 mm, and 30 slices. The acquired images were imported into ITK-SNAP software. Regions of interest (ROIs), including the lateral ventricle, hippocampus, cortex, and corpus callosum, were manually delineated slice by slice, followed by three-dimensional reconstruction and volumetric quantification.

Diffusion tensor imaging: DTI data were acquired in the coronal plane using a single-shot spin-echo echo-planar imaging sequence. The scanning parameters were as follows: TR = 6,752 ms, TE = 22 ms, FOV = 3.5 cm × 3.5 cm, matrix = 128 × 128, number of excitations = 1, and slice thickness = 1.00 mm. Diffusion gradients were applied in 30 directions, with *b*-values of 0 and 1,000 s/mm^2^. Images were post-processed using the corresponding workstation software to extract and calculate fractional anisotropy (FA) values in key ROIs, including the genu of the corpus callosum, cingulum, dorsal hippocampus, and thalamic core region, to assess the microstructural integrity of neural fibers.

### Terminal anesthesia and sample collection

At the terminal sampling time point, rats were deeply anesthetized with isoflurane delivered in medical oxygen until the absence of pedal withdrawal and corneal reflexes. For serum assays, abdominal aortic blood was collected under deep anesthesia, after which rats were euthanized by exsanguination and brain tissues were rapidly harvested. For histological and ultrastructural analyses, rats were transcardially perfused under deep anesthesia with normal saline followed by the appropriate fixative. Death was confirmed by cessation of heartbeat and respiration before tissue collection.

### Luxol fast blue staining

Sections were first deparaffinized in xylene three times for 10 min each, and then rehydrated through graded ethanol, including absolute ethanol I, absolute ethanol II, and 95% ethanol, for 5 min each. Sections were incubated in LFB staining solution overnight at room temperature. The next day, excess staining solution was removed with 95% ethanol, and sections were differentiated in Luxol differentiation solution for 3 s. Sections were then rinsed with tap water and examined under a microscope to monitor differentiation. Finally, sections were dehydrated through graded ethanol (95% ethanol, absolute ethanol I, and absolute ethanol II, 5 min each), cleared in xylene I and II for 10 min each, mounted with neutral resin, and observed under a light microscope to assess myelin integrity.

### Transmission electron microscopy

To observe ultrastructural changes in corpus callosum nerve fibers and myelin sheaths, rats were transcardially perfused with normal saline and pre-cooled 2.5% glutaraldehyde fixative. Brain tissue was rapidly removed on ice, and approximately 1-mmł tissue blocks were precisely dissected from the corpus callosum. Tissue blocks were fully immersed in 2.5% glutaraldehyde and stored overnight at 4 °C in the dark. Samples were then rinsed with 0.1 M phosphate-buffered saline (PBS) and post-fixed in 1% osmium tetroxide for 2 h. After dehydration through graded ethanol and acetone, samples were embedded in epoxy resin and polymerized. Ultrathin sections approximately 60–80 nm thick were cut with an ultramicrotome and mounted on copper grids. Sections were double-stained with 2% uranyl acetate and lead citrate, and high-resolution images of myelin lamellae were acquired under a transmission electron microscope (JEOL JEM-1400). TEM was used for qualitative assessment of myelin ultrastructural morphology. Because images were not acquired using a systematic morphometric sampling protocol, retrospective quantitative analysis of parameters such as the g-ratio was not performed.

### ELISA and biochemical assays

Claudin-5 (Jiangsu Jingmei Biotechnology, China; Cat. No. JM-11287R1), ZO-1 (Jiangsu Jingmei Biotechnology; Cat. No. JM-01792R1), Iba1 (Ke Aibo Biotechnology, China; Cat. No. CB13921-Ra), GFAP (Jiangsu Jingmei Biotechnology; Cat. No. JM-01781R1), MBP (Jiangsu Jingmei Biotechnology; Cat. No. JM-01870R1), NfL (Ke Aibo Biotechnology; Cat. No. CB13667-Ra), and NSE (Jiangsu Jingmei Biotechnology; Cat. No. JM-01476R1) were measured using rat-specific ELISA kits according to the manufacturers’ instructions. MDA levels were determined using a Lipid Peroxidation MDA Assay Kit (Beyotime Biotechnology, Shanghai, China; Cat. No. S0131S), and total SOD activity was measured using a Total SOD Assay Kit with WST-8 (Beyotime Biotechnology; Cat. No. S0101S).

Brain tissue ELISA: Hippocampal, frontal cortical, and corpus callosum tissues were rapidly dissected. The tissues were perfused and rinsed with ice-cold PBS (4 °C) to remove residual vascular blood, accurately weighed, and mechanically cut into approximately 1-mm^3^ fragments. Ice-cold PBS was added at a mass-to-volume ratio of 1:9, and tissues were homogenized intermittently on ice using a tissue grinder at 600 rpm (3 × 15-s pulses). Homogenates were centrifuged at 5,000×*g* for 10 min at 4 °C, and the supernatants were collected as tissue lysates. According to the kit instructions, Claudin-5, ZO-1, Iba1, GFAP, MDA, and SOD were quantified in the hippocampus and frontal cortex, and MBP and NfL were quantified in the corpus callosum. Standard curve wells were included for quality control.

Serum ELISA: Rats were randomly selected from each group for abdominal aortic blood collection. Blood samples were allowed to stand for 30 min and then centrifuged at 3,000×*g* for 15 min at 4 °C. The upper serum fraction was collected, aliquoted, and stored at −80 °C until analysis. Serum NSE and NfL concentrations were measured strictly according to the kit protocols. Standard curve wells were included to ensure that measurements fell within the linear detection range. Background interference was corrected using blank wells, and actual concentrations were calculated according to the standard curve equation.

### Statistical analysis

All data were analyzed and graphed using GraphPad Prism 10.4.4. Continuous variables are presented as mean ± standard deviation (SD). The individual animal was treated as the experimental and statistical unit. Technical replicates or repeated measurements obtained from the same animal were averaged before group-level statistical analysis.

Before statistical testing, normality was assessed using the Shapiro–Wilk test for each quantitative outcome. No dataset included in the final inferential analyses showed a significant deviation from normality at *P* < 0.05; therefore, parametric statistical tests were used for all quantitative outcomes, and no non-parametric tests were required. Homogeneity of variance was also evaluated before single-time-point multi-group comparisons.

Escape latency during days 1–5 of the Morris water maze and longitudinal blood pressure measurements were analyzed using two-way repeated-measures ANOVA, with group as the between-subjects factor and training day or time as the within-subjects factor, followed by Turkey’s multiple-comparisons test when appropriate. Single-time-point multi-group outcomes, including probe-trial indices, open field test parameters, balance beam test parameters, MRI/DTI measurements, and molecular assay results, were analyzed using one-way ANOVA followed by Turkey’s multiple-comparisons test.

For graphical clarity, statistical significance is indicated using symbol-based notation in the main figures. Exact multiplicity-adjusted *P* values, omnibus test statistics, effect estimates, 95% confidence intervals, and complete pairwise-comparison results are provided in Supplementary Table 1. All tests were two-sided, and *P* < 0.05 was considered statistically significant.

## Results

### Induction of the composite-risk-factor CSVD rat model and dynamic blood pressure changes

**FIGURE 1:**
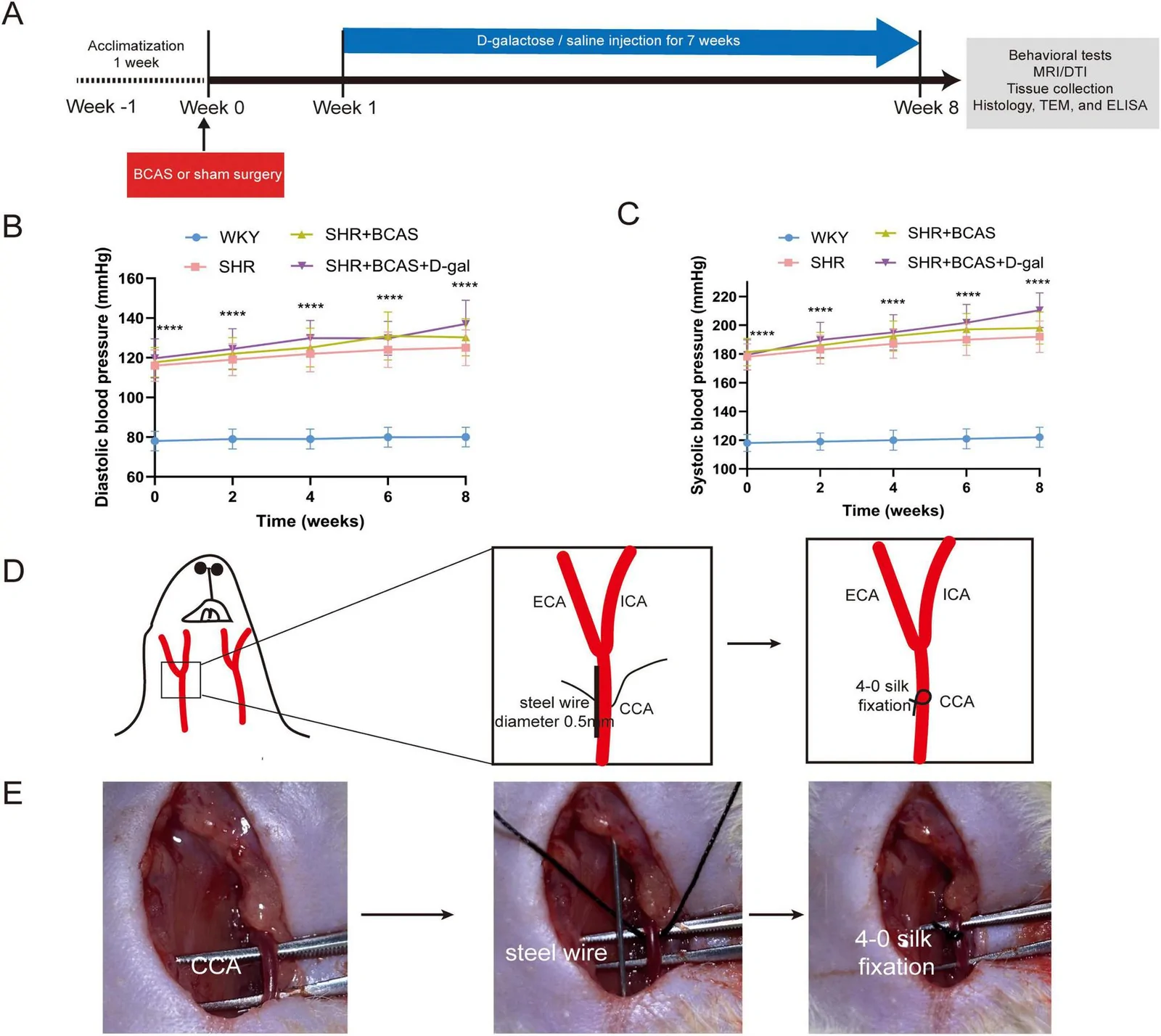
Induction of the composite-risk-factor CSVD rat model, dynamic blood pressure changes, and schematic of the BCAS procedure. **(A)** Experimental timeline. After 1 week of acclimatization, rats underwent BCAS or sham surgery at week 0. D-galactose or saline was administered from week 1 for 7 consecutive weeks. Behavioral testing, MRI/DTI scanning, tissue collection, and pathological and molecular analyses were performed after week 8. **(B)** Changes in diastolic blood pressure over time. **(C)** Changes in systolic blood pressure over time. **(D)** Schematic of key anatomical structures and procedural steps in BCAS surgery, showing the external carotid artery, internal carotid artery, and common carotid artery, as well as stenosis induced using a 0.5-mm steel wire and 4-0 silk ligation. **(E)** Representative intraoperative photographs showing CCA exposure, steel-wire-assisted positioning, and 4-0 silk ligation. BCAS, bilateral common carotid artery stenosis; CCA, common carotid artery; ECA, external carotid artery; ICA, internal carotid artery; D-gal, D-galactose. Data are presented as mean ± SD. Longitudinal diastolic and systolic blood pressure data were analyzed using two-way repeated-measures ANOVA, with group as the between-subjects factor and time as the within-subjects factor, followed by Turkey’s multiple-comparisons test. For graphical clarity, a single set of asterisks is shown above each time point when all displayed comparisons between WKY and the three hypertensive groups (SHR, SHR+BCAS, and SHR+BCAS+D-gal) reached the same significance threshold. ****P < 0.0001. Exact multiplicity-adjusted *P* values, omnibus test statistics, effect estimates, 95% confidence intervals, and sample sizes at each time point are provided in Supplementary Table 1.

**FIGURE 2:**
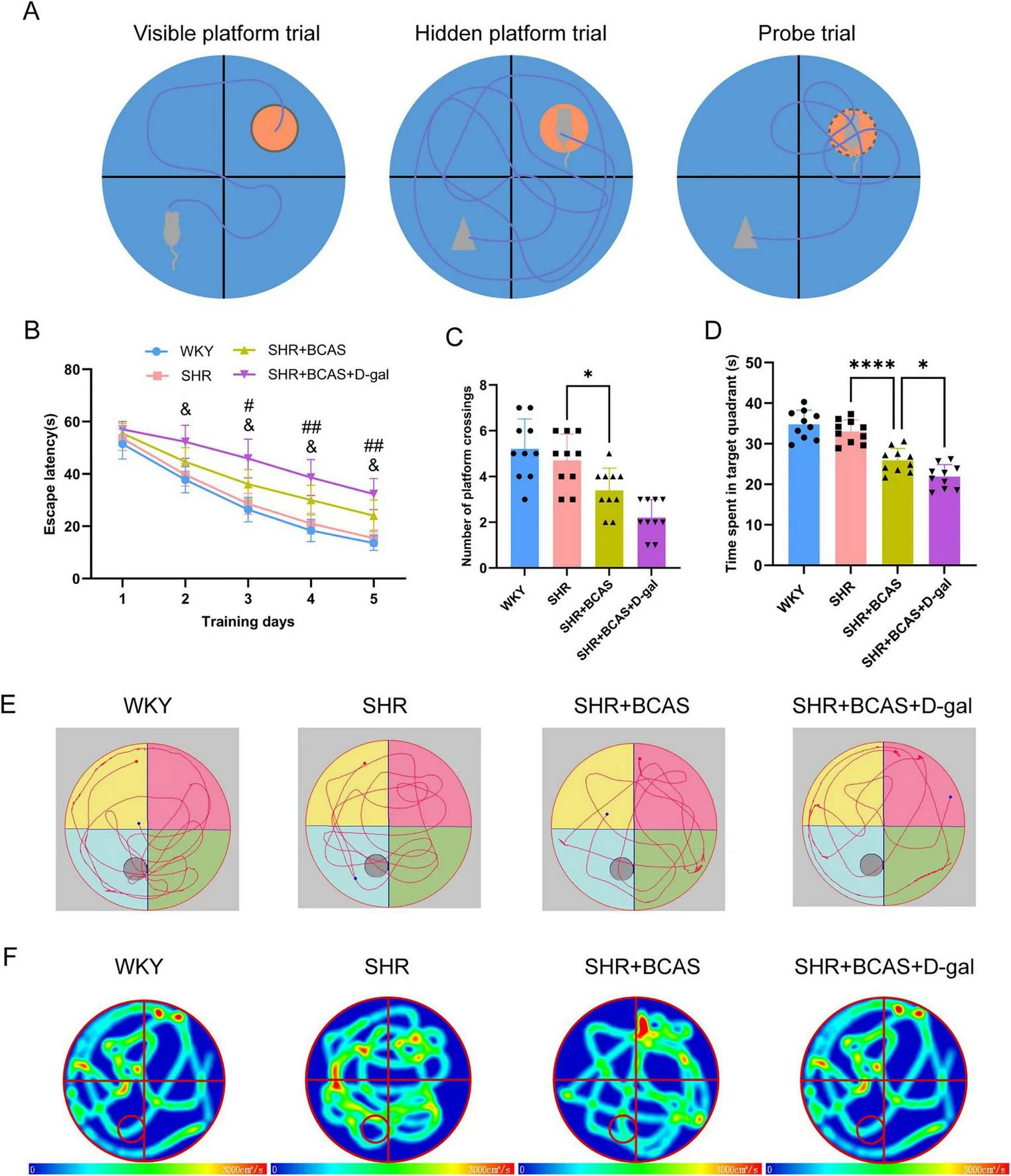
The composite-risk-factor CSVD model aggravates spatial learning and memory impairment. **(A)** Schematic of the Morris water maze procedure. **(B)** Escape latency during the 5-day place navigation training phase. **(C)** Number of crossings over the former platform location in the probe trial after platform removal. **(D)** Time spent in the target quadrant after platform removal. **(E)** Representative swimming trajectories of rats in each group during the probe trial. **(F)** Representative heatmaps of swimming paths showing the spatial distribution of search intensity. Data are presented as mean ± SD, *n* = 10 animals/group. Escape latency during training days 1–5 was analyzed using two-way repeated-measures ANOVA, with group as the between-subjects factor and training day as the within-subjects factor, followed by Turkey’s multiple-comparisons test. Probe-trial indices, including platform crossings and target-quadrant dwell time, were analyzed using one-way ANOVA followed by Turkey’s multiple-comparisons test. For graphical clarity, significance is indicated using asterisk-based notation in the figure. In the figure, asterisks indicate comparisons with the WKY group, hash symbols indicate SHR vs. SHR+BCAS, and ampersands indicate SHR+BCAS vs. SHR+BCAS+D-gal. *P < 0.05 and ****P < 0.0001; ^#^*P* < 0.05 and ^##^*P* < 0.01; ^&^*P* < 0.05. Exact multiplicity-adjusted *P* values, omnibus test statistics, effect estimates, 95% confidence intervals, and complete pairwise-comparison results are provided in Supplementary Table 1.

**FIGURE 3:**
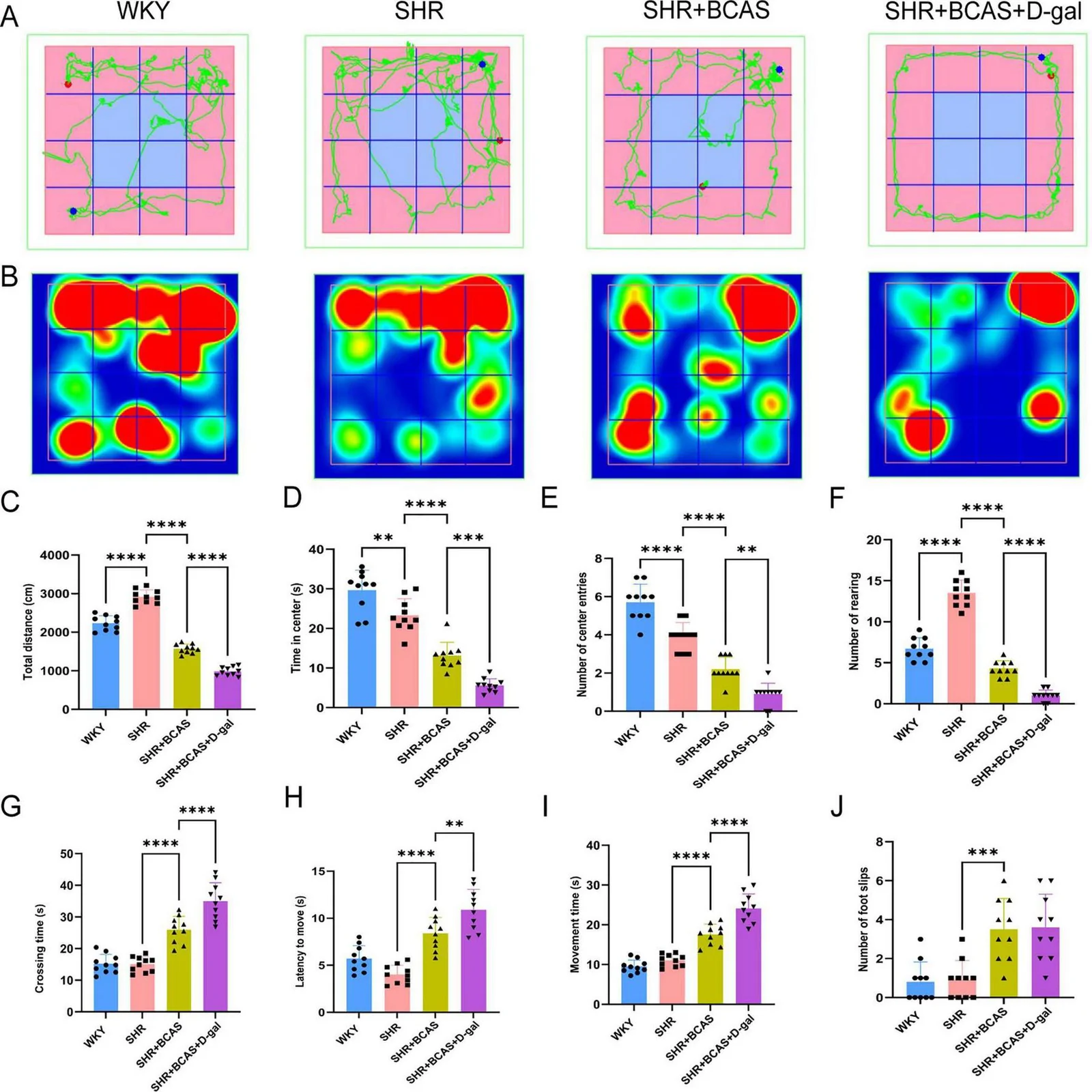
Open field and balance beam tests reveal alterations in locomotor/exploratory activity and balance-related performance following exposure to composite risk factors. **(A)** Representative activity trajectories in the open field test. **(B)** Representative heatmaps of open field activity reflecting avoidance of the central zone. **(C–F)** Quantitative indices from the open field test, including **(C)** total distance traveled, **(D)** time spent in the central zone, **(E)** number of central-zone entries, and **(F)** number of rearings. **(G–J)** Quantitative indices from the balance beam test, including **(G)** crossing time, **(H)** number of foot slips, **(I)** active movement time, and **(J)** waiting/stopping time. Each quantitative outcome was analyzed separately using one-way ANOVA followed by Turkey’s multiple-comparisons test. For graphical clarity, significance is indicated using asterisk-based notation in the figure (**P < 0.01, ***P < 0.001, and ****P < 0.0001). Exact multiplicity-adjusted *P* values, omnibus test statistics, effect estimates, 95% confidence intervals, and complete pairwise-comparison results are provided in Supplementary Table 1.

During the experiment, no rats died in the WKY or SHR groups, whereas two rats died in the SHR+BCAS group and two rats died in the SHR+BCAS+D-gal group during the modeling period. Therefore, 10 animals remained available in each BCAS-containing group for behavioral testing. To maintain balanced group sizes for behavioral comparisons, 10 animals from each of the WKY and SHR groups were included in the behavioral cohort. The two remaining animals in the WKY and SHR groups were not excluded because of adverse health events, missing behavioral data, or data-quality concerns. Thus, behavioral analyses in Figures 2, 3 were performed using *n* = 10 animals per group. The final numbers of animals included in MRI, ELISA, LFB staining, and TEM analyses are shown in the corresponding figure legends. In this study, a composite-risk-factor CSVD rat model was induced by combining hypertension, chronic cerebral hypoperfusion, and aging-related injury. The experimental procedure is shown in Figure 1A. After 1 week of acclimatization, rats underwent BCAS or sham surgery at week 0. Beginning 1 week after surgery, rats received D-galactose or an equal volume of normal saline for 7 consecutive weeks. After week 8, behavioral testing, MRI/DTI scanning, tissue sampling, and pathological and molecular analyses were performed.

Blood pressure monitoring showed that diastolic and systolic blood pressure remained relatively low and stable in the WKY group throughout the experimental period. Compared with WKY rats, SHR, SHR+BCAS, and SHR+BCAS+D-gal rats showed persistently elevated diastolic and systolic blood pressure at all time points. As the experiment progressed, blood pressure showed a mild upward trend in the SHR+BCAS and SHR+BCAS+D-gal groups, with the SHR+BCAS+D-gal group showing higher blood pressure levels at later time points. These findings indicate that the hypertensive background was maintained during composite model establishment and coexisted with chronic hypoperfusion and aging-related intervention to form a multi-risk-factor state (Figures 1B, C).

The BCAS surgical procedure is shown in Figures 1D, E. During surgery, the bilateral CCAs were fully exposed, and the CCA and its branches were separated. A 0.5-mm steel wire was used as a sizing aid, and the CCA was fixed using 4-0 silk sutures to establish bilateral carotid stenosis. This method induced chronic cerebral hypoperfusion under a hypertensive SHR background, providing an experimental basis for evaluating CSVD-like changes induced by composite risk factors.

### The composite-risk-factor CSVD model further aggravates spatial learning and memory deficits

To determine the effect of multiple risk factors on cognitive function, spatial learning and memory were evaluated using the MWM. During the place navigation test (training days 1–5), escape latency gradually decreased across training days in all groups, indicating acquisition of spatial learning. However, compared with the WKY group, SHR rats showed significantly prolonged escape latency. Notably, after superimposition of chronic cerebral hypoperfusion and aging-related stress, learning performance was further impaired, with the SHR+BCAS+D-gal group showing the longest escape latency across training time points (Figure 2B). In the probe trial on day 6 after platform removal, WKY rats spent more time in the target quadrant and crossed the former platform location more frequently, indicating better retention of spatial memory. By contrast, both indices were reduced in SHR rats, and the cognitive deficit progressively worsened with the introduction of ischemic and aging-related factors. The SHR+BCAS+D-gal group had the fewest platform crossings (Figure 2C) and the shortest time spent in the target quadrant (Figure 2D), indicating the most severe memory impairment. Representative swimming traces and heatmaps further showed that rats exposed to composite risk factors adopted random or peripheral searching strategies and failed to focus effectively on the target area (Figures 2E, F).

### Open field and balance beam tests reveal alterations in locomotor/exploratory activity and balance-related performance

In addition to cognitive impairment, neurological disease often causes abnormalities in emotional behavior and motor coordination. The open field test showed that, compared with WKY rats, SHR rats exhibited changes in total distance traveled and central-zone activity, suggesting a possible tendency toward anxiety-like behavior. After the addition of chronic cerebral hypoperfusion and aging-related stress, spontaneous locomotion and exploratory behavior were further reduced in the SHR+BCAS and SHR+BCAS+D-gal groups, as reflected by decreased total distance traveled, reduced time spent in and entries into the central zone, and fewer rearings. The balance beam test further showed that, as pathological factors were added, beam crossing time was gradually prolonged and the number of foot slips increased. In particular, SHR+BCAS+D-gal rats required markedly longer time to complete the task and exhibited more pauses, waiting, and hesitation during locomotion, indicating further impairment of motor execution efficiency and balance regulation.

### T2-weighted MRI demonstrates structural atrophy in core cognitive and white matter regions induced by composite risk factors

**FIGURE 4:**
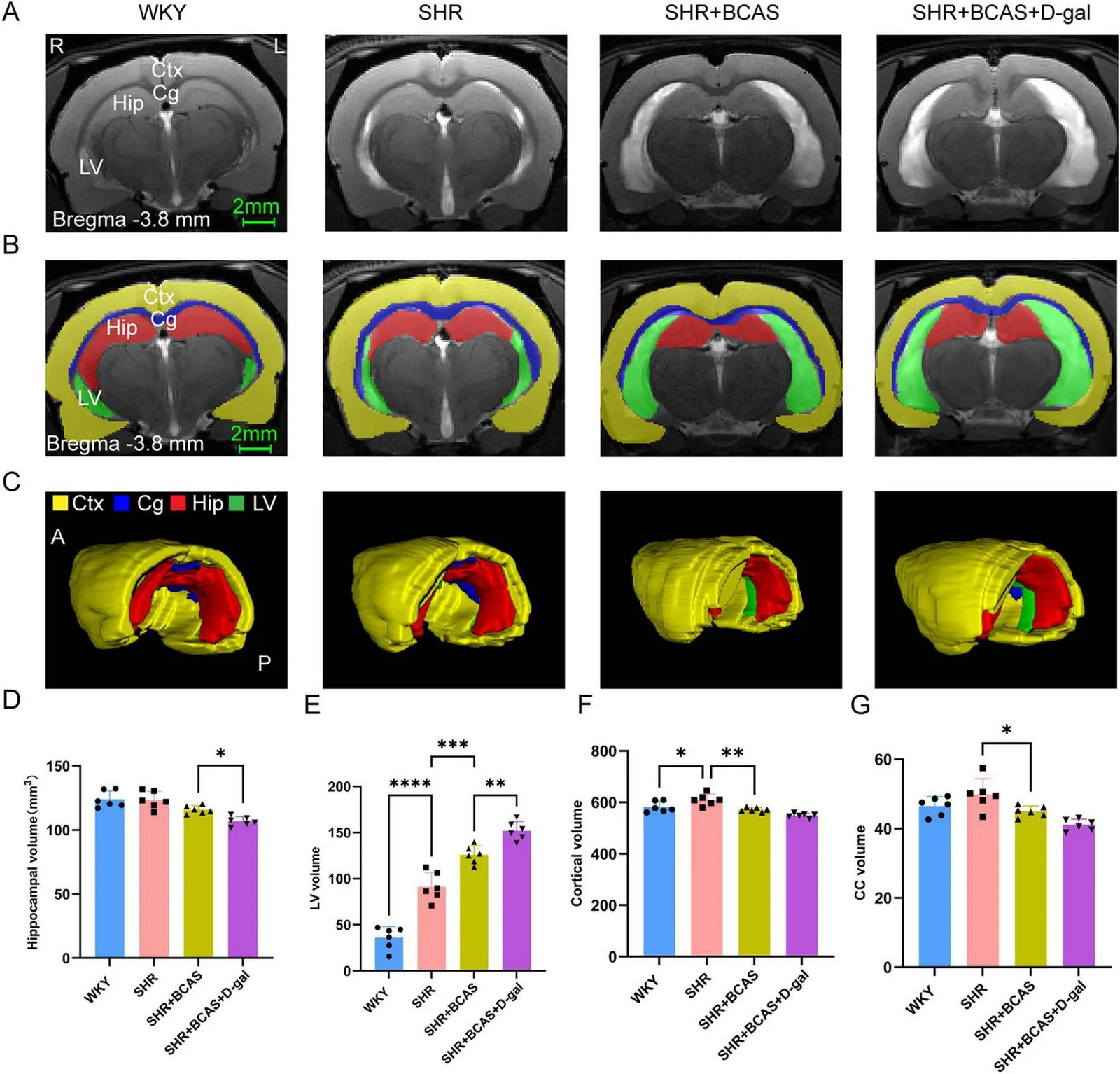
T2-weighted MRI demonstrates prominent effects of composite risk factors on core brain region volumes. **(A)** Representative raw axial T2-weighted MRI images of the rat brain. Ctx, cortex; Cg, cingulum; Hip, hippocampus; LV, lateral ventricle; Bregma, –3.8 mm. **(B)** Representative pseudo-color images of manual ROI segmentation using ITK-SNAP. **(C)** Representative lateral spatial images of three-dimensional reconstructions of target brain regions. **(D–G)** Quantitative volumetric analysis of core brain regions, including **(D)** hippocampal volume, **(E)** lateral ventricle volume, **(F)** cortical volume, and **(G)** corpus callosum volume. Data are presented as mean ± SD, *n* = 6. Hippocampal, lateral ventricle, cortical, and corpus callosum volumes were analyzed separately using one-way ANOVA followed by Turkey’s multiple-comparisons test. For graphical clarity, significance is indicated using asterisk-based notation in the figure (*P < 0.05, **P < 0.01, ***P < 0.001, and ****P < 0.0001). Exact multiplicity-adjusted *P* values, omnibus test statistics, effect estimates, 95% confidence intervals, and complete pairwise-comparison results are provided in Supplementary Table 1.

To clarify macroscopic structural changes *in vivo*, high-resolution T2-weighted MRI was used for three-dimensional reconstruction and volumetric quantification of core brain regions. Representative T2-weighted MRI images, ROI segmentation, and three-dimensional reconstruction of brain structures are shown in Figures 4A–C. Compared with WKY rats, SHR rats already showed significant compensatory enlargement of the lateral ventricles (LVs; Figure 4E), indicating chronic compression and atrophy of surrounding brain tissue induced by long-term blood pressure burden. With the addition of cerebral hypoperfusion and aging-related stress (SHR+BCAS and SHR+BCAS+D-gal), target brain region atrophy showed a progressive worsening trend. The hippocampus and cortex, which are key gray matter regions responsible for memory and higher executive function, showed further volume reduction as risk factors accumulated, with the most pronounced decreases observed in the SHR+BCAS+D-gal group (Figures 4D, F). Similarly, the corpus callosum (CC), the largest white matter fiber bundle, showed significant volume reduction and thinning (Figure 4G). Lateral ventricle volume reached its maximum under the combined influence of all three risk factors (Figure 4E). Three-dimensional reconstruction further demonstrated progressive aggravation of structural changes in core gray and white matter regions with the combined exposure to hypertension, chronic cerebral hypoperfusion, and D-gal stimulation. These results suggest that composite risk factors induce prominent structural abnormalities in core cognitive-related brain regions and white matter.

### DTI-MRI indicates that composite risk factors disrupt neural fiber integrity in core white matter regions

**FIGURE 5:**
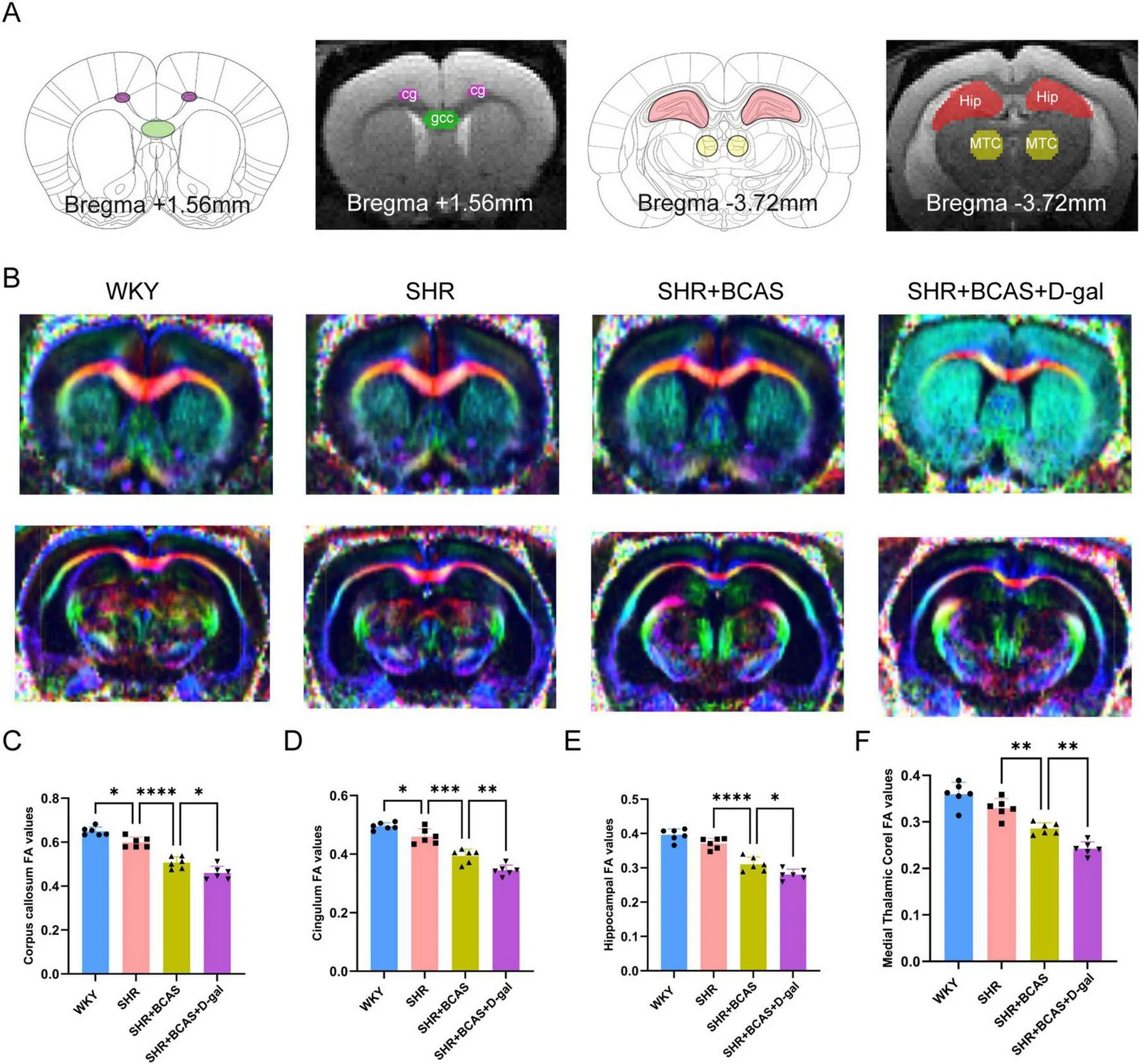
DTI indicates that composite risk factors disrupt neural fiber integrity in core white matter regions. **(A)** Standard ROI segmentation diagrams based on a rat brain atlas and T2-weighted images at Bregma 1.56 mm and –3.72 mm. **(B, C)** Representative FA orientation color maps derived from DTI: **(B)** FA maps of the genu of the corpus callosum (gcc) and cingulum (Cg); **(C)** FA maps of the dorsal hippocampus (Hip) and thalamic core region (MTC). **(C–F)** Quantitative analysis of FA values in core ROIs, including **(C)** gcc FA, **(D)** Cg FA, **(E)** dorsal hippocampus FA, and **(F)** MTC FA. Data are presented as mean ± SD, *n* = 6. Regional FA values were analyzed separately using one-way ANOVA followed by Turkey’s multiple-comparisons test. For graphical clarity, significance is indicated using asterisk-based notation in the figure (*P < 0.05, **P < 0.01, ***P < 0.001, and ****P < 0.0001). Exact multiplicity-adjusted *P* values, omnibus test statistics, effect estimates, 95% confidence intervals, and complete pairwise-comparison results are provided in Supplementary Table 1.

After confirming macroscopic brain atrophy, DTI was used to quantitatively evaluate microstructural white matter fiber integrity by measuring FA. Representative ROI locations used for DTI analysis are shown in Figure 5A. Reduced FA generally indicates demyelination or impaired axonal integrity. Compared with WKY rats, SHR rats showed a downward trend in FA values in the genu of the corpus callosum (gcc), cingulum (Cg), dorsal hippocampus (Hip), and thalamic core region (MTC).

When chronic cerebral hypoperfusion and aging-related stress were added, white matter microstructural injury was further aggravated. FA orientation color maps showed that fiber tracts in the corpus callosum and corona radiata became disorganized and sparse in the SHR+BCAS and SHR+BCAS+D-gal groups, with visibly attenuated color signals (Figures 5B, C). Quantitative analysis confirmed that the SHR+BCAS+D-gal group had the lowest FA values in the gcc (Figure 5C), Cg (Figure 5D), dorsal hippocampus (Figure 5E), and MTC (Figure 5F). These results indicate that hypertension, chronic cerebral hypoperfusion, and aging-related stress jointly impair white matter microstructural integrity in CSVD-related key brain regions.

### Histopathology and serum biomarkers indicate that composite risk factors aggravate white matter and axonal injury

**FIGURE 6:**
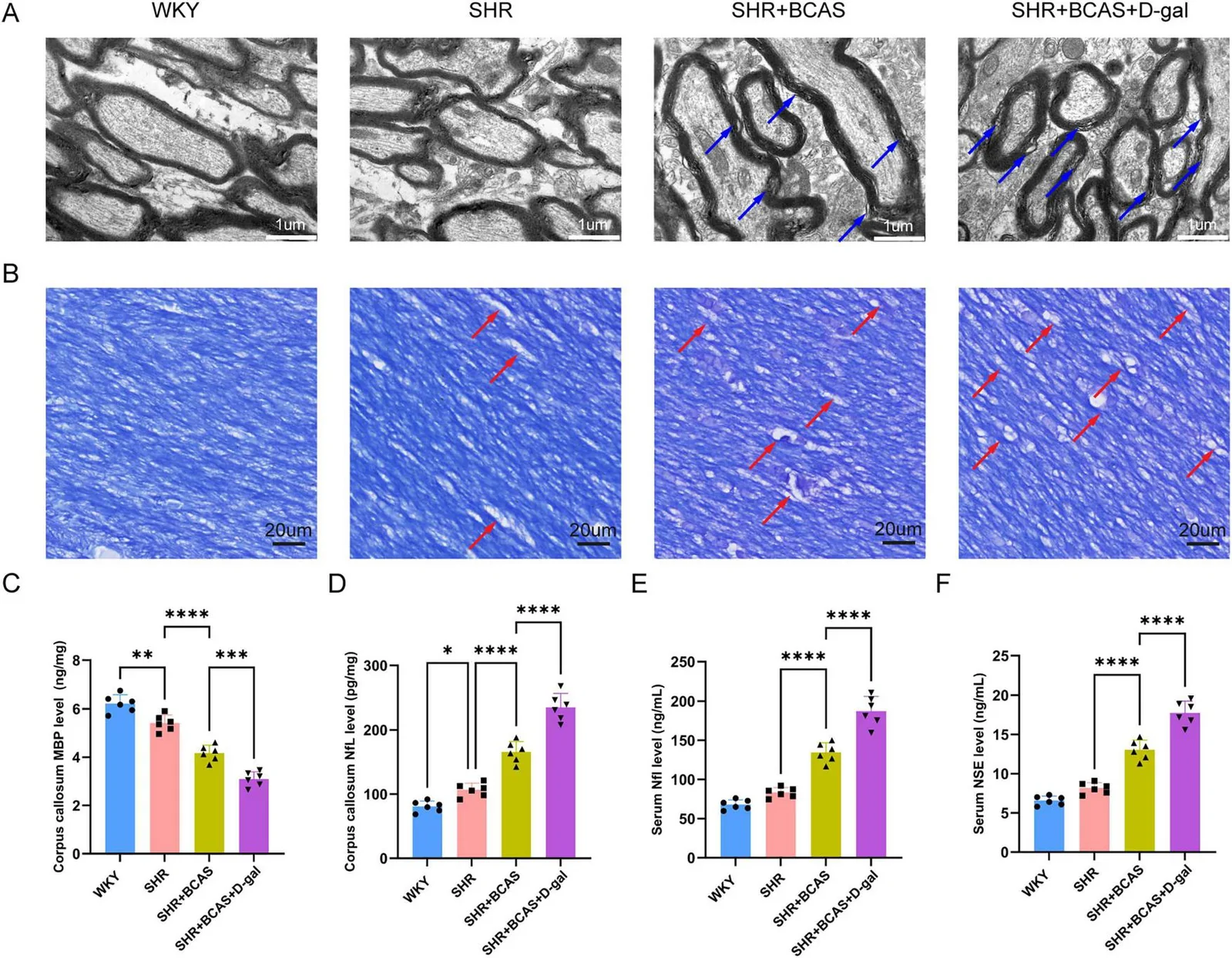
Histopathology and serum biomarkers confirm that composite risk factors aggravate white matter injury. **(A)** Representative TEM images showing myelin sheath ultrastructure of nerve fibers in the corpus callosum; blue arrows indicate areas of myelin lamellar loosening, irregularity, or deformation. **(B)** Representative LFB-stained images showing overall nerve fiber arrangement and myelin integrity in the corpus callosum; red arrows indicate areas of reduced LFB staining and disrupted fiber organization. Scale bars: 1 μm in TEM images and 20 μm in LFB-stained images. **(C)** MBP levels in the corpus callosum. **(D)** NfL levels in the corpus callosum. **(E)** Serum NfL levels. **(F)** Serum NSE levels. Abbreviations: MBP, myelin basic protein; NfL, neurofilament light chain; NSE, neuron-specific enolase; TEM, transmission electron microscopy; LFB, Luxol fast blue. TEM and LFB observations were performed in *n* = 3 animals/group, whereas molecular measurements were performed in *n* = 6 animals/group. Molecular data are presented as mean ± SD. For graphical clarity, significance is indicated using asterisk-based notation in the figure (*P < 0.05, **P < 0.01, ***P < 0.001, and ****P < 0.0001).

The white matter microstructural abnormalities detected by DTI were further evaluated at the histopathological, ultrastructural, and protein-expression levels. TEM showed compact myelin sheaths with regularly arranged lamellae and clear boundaries in the corpus callosum of WKY rats. In contrast, SHR rats and rats exposed to additional hypoperfusion and aging-related factors exhibited progressively more evident myelin lamellar loosening, irregular arrangement, and deformation, with the most prominent abnormalities observed in the SHR+BCAS+D-gal group (Figure 6A). LFB staining showed lighter blue staining, looser nerve-fiber arrangement, and focal disruption in the model groups, particularly in the SHR+BCAS+D-gal group (Figure 6B). At the molecular level, the stepwise reduction in myelin basic protein (MBP) in the corpus callosum further supported impaired myelin integrity (Figure 6C). Meanwhile, neurofilament light chain (NfL), a core marker of axonal cytoskeletal injury, was markedly elevated in the corpus callosum (Figure 6D). Notably, increased serum NfL and neuron-specific enolase (NSE) levels further indicated axonal and neuronal injury-related changes in model rats, with the highest levels observed in the SHR+BCAS+D-gal group (Figures 6E, F). Together, these findings indicate that the three-factor model is associated with myelin ultrastructural abnormalities and increased biomarkers related to axonal and neuronal injury.

### Composite risk factors induce abnormalities in BBB-associated proteins, neuroinflammation, and oxidative stress

**FIGURE 7:**
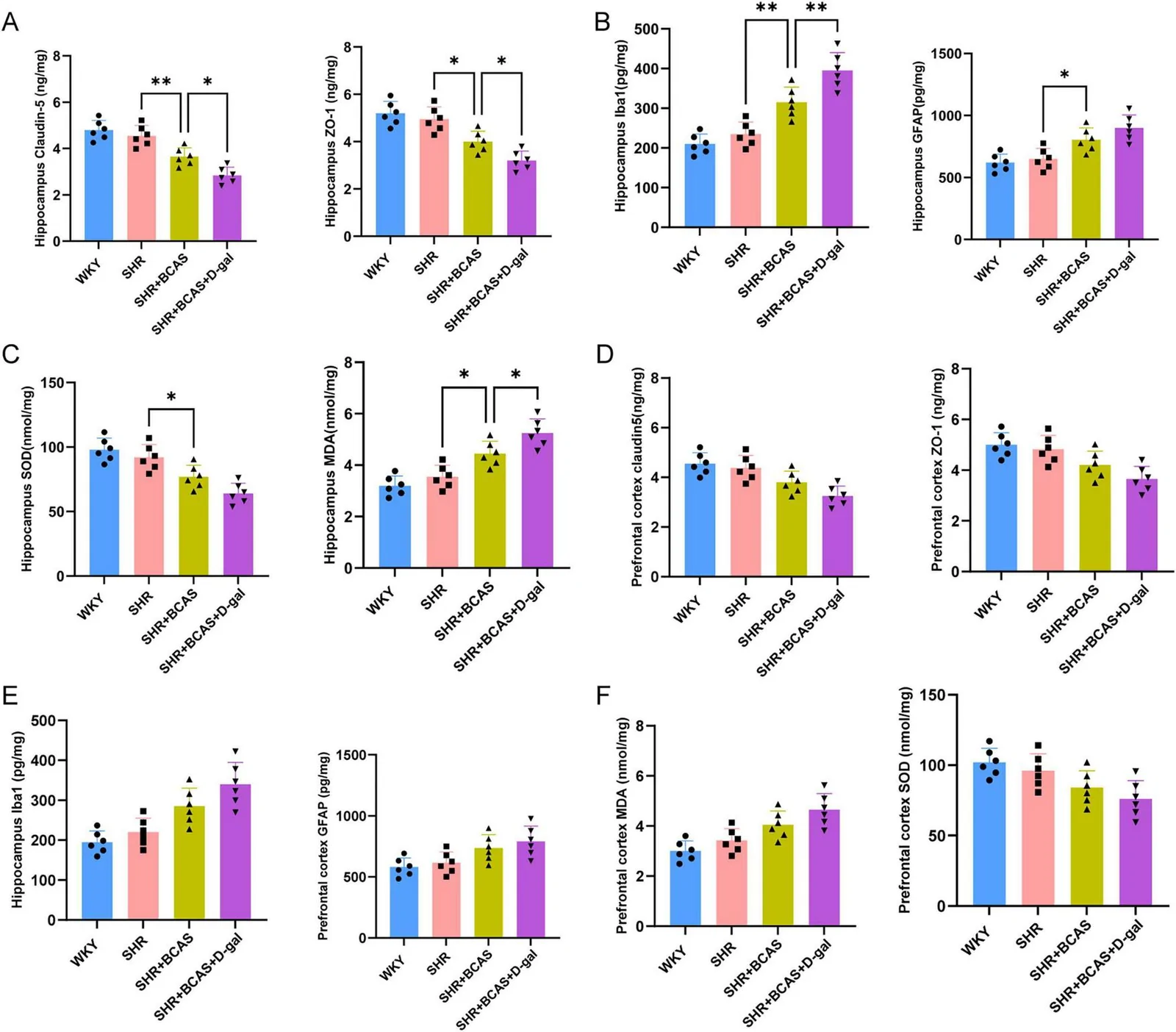
The composite-risk-factor model induces abnormalities in BBB-associated proteins, neuroinflammation, and oxidative stress markers in the hippocampus and frontal cortex. **(A–C)** Molecular assay results in hippocampal tissue, including **(A)** BBB tight-junction proteins Claudin-5 and ZO-1, **(B)** neuroinflammation-related markers Iba1 and GFAP, and **(C)** oxidative stress-related markers MDA and SOD. **(D–F)** Molecular assay results in frontal cortical tissue, including **(D)** Claudin-5 and ZO-1, **(E)** Iba1 and GFAP, and **(F)** MDA and SOD. Data are presented as mean ± SD, *n* = 6/group. Abbreviations: BBB, blood-brain barrier; GFAP, glial fibrillary acidic protein; Iba1, ionized calcium-binding adapter molecule 1; MDA, malondialdehyde; SOD, superoxide dismutase. Each molecular or biochemical marker was analyzed separately using one-way ANOVA followed by Turkey’s multiple-comparisons test. For graphical clarity, significance is indicated using asterisk-based notation in the figure (*P < 0.05 and **P < 0.01). Exact multiplicity-adjusted *P* values, omnibus test statistics, effect estimates, 95% confidence intervals, and complete pairwise-comparison results are provided in Supplementary Table 1.

To further evaluate the effects of composite risk factors on microenvironmental homeostasis in core cognition-related brain regions, we measured tight-junction proteins, neuroinflammatory markers, and oxidative stress-related indicators in the hippocampus and frontal cortex (Figure 7).

ELISA showed that, compared with WKY rats, model rats exhibited decreased Claudin-5 and ZO-1 levels in the hippocampus and frontal cortex, with the most pronounced decreases observed in the SHR+BCAS+D-gal group. These results suggest that composite risk factors may impair tight-junction protein expression and BBB structural integrity. Concurrently, Iba1 and GFAP levels were elevated in the hippocampus and frontal cortex, indicating enhanced microglia- and astrocyte-related inflammatory responses. Oxidative stress assays showed increased MDA levels and decreased SOD activity in model rats, and these changes were more evident in the SHR+BCAS+D-gal group. Together, these results indicate that chronic cerebral hypoperfusion and D-gal stimulation superimposed on a hypertensive background can induce abnormalities in BBB-related protein expression, enhanced neuroinflammation, and oxidative stress imbalance, which may contribute to white matter injury and behavioral abnormalities in this composite model.

## Discussion

In the present study, we established a composite CSVD rat model by combining BCAS surgery and D-gal-induced aging-related stress on the background of spontaneous hypertension in SHRs. The results showed that, as risk factors accumulated, model rats developed more prominent spatial learning and memory deficits, reduced locomotor and exploratory activity and altered central-zone behavior, and impaired motor coordination and balance. T2-weighted MRI and DTI further demonstrated structural alterations in core brain regions and reduced white matter fiber integrity. Histopathological and molecular assays showed that this composite model was accompanied by corpus callosum myelin disruption, decreased MBP expression, elevated NfL and NSE levels, possible disruption of BBB-related structural integrity in the hippocampus and frontal cortex, glial activation, and oxidative stress imbalance. Previous studies have shown that CSVD is not only an important pathological basis of vascular cognitive impairment, but is also frequently accompanied by motor dysfunction, impaired balance, and emotional/behavioral abnormalities (Markus and de Leeuw, 2023). Hypertension-related CSVD frequently involves white matter, deep gray matter, and cortical-subcortical networks, and is closely associated with cognitive decline and structural brain damage (Csiszar et al., 2024). Together, these findings demonstrate progressive behavioral, neuroimaging, histopathological, and molecular alterations across the sequential model groups. The development and progression of CSVD are usually not driven by a single factor, but by the long-term interaction of vascular risk factors, perfusion abnormalities, and age-related degenerative changes. Hypertension and aging are considered the most important risk factors for sporadic CSVD and can promote white matter damage and cognitive decline through arteriolosclerosis, endothelial dysfunction, reduced vascular compliance, and impaired regulation of the cerebral microcirculation (Washida et al., 2019; Hainsworth et al., 2024). Chronic cerebral hypoperfusion causes persistent ischemia, hypoxia, and energy metabolic dysfunction in brain tissue, and is an important pathological basis for white matter lesions and vascular cognitive impairment (Washida et al., 2019). In addition, D-gal-induced aging models can partially mimic age-related cognitive decline, oxidative stress, and neurochemical changes in the brain (Sadigh-Eteghad et al., 2017). Therefore, SHR mainly provides a chronic hypertensive and cardiovascular-risk background with relatively mild early CSVD-like features, whereas BCAS primarily introduces chronic cerebral hypoperfusion and D-gal stimulation contributes aging-related vulnerability. Each factor alone is insufficient to reproduce the multifactorial, chronic, and multisystem nature of clinical CSVD.

An additional point concerns the blood-pressure response after BCAS. Although systemic blood pressure might theoretically increase as a compensatory response to cerebral hypoperfusion, BCAS did not induce a robust additional elevation of tail-cuff blood pressure beyond the hypertensive SHR background in the present study. Several factors may explain this observation. First, SHRs already exhibited sustained hypertension, which may have limited the dynamic range for further systemic blood-pressure elevation. Second, BCAS produces a local mechanical restriction of carotid blood flow, and compensation for reduced cerebral perfusion may occur mainly through local cerebrovascular autoregulation, vasodilation, collateral circulation, and metabolic or neurovascular responses rather than through a sustained increase in peripheral systemic blood pressure. Third, tail-cuff blood-pressure measurements at scheduled time points may not capture transient acute pressor responses after carotid stenosis. Because cerebral blood flow, collateral circulation, autonomic activity, and neurohumoral responses were not directly measured, the present study cannot determine whether compensatory mechanisms were activated after BCAS. Future studies combining longitudinal blood-pressure monitoring with cerebral perfusion imaging or laser speckle flowmetry would be useful for clarifying the relationship between systemic blood pressure and cerebral hypoperfusion in this model.

Recent work by Meng et al. (2024) showed that hypertension, aging, and cerebral hypoperfusion can be integrated into a single rat model, providing important evidence supporting the translational relevance of this three-factor framework. However, their study did not fully address several phenotypic dimensions that are critical for evaluating CSVD-related neural injury, including quantitative 7.0-T DTI-FA assessment of white matter microstructural integrity, TEM-based ultrastructural validation of myelin lamellar injury, serum neural injury biomarkers such as NfL and NSE, and dedicated assessment of fine motor coordination and balance using the balance beam test. Thus, an important knowledge gap remains regarding whether this composite-risk-factor model can be systematically characterized across behavioral, neuroimaging, histopathological, ultrastructural, and peripheral biomarker levels.

The present study extends this previous work by providing a comprehensive multimodal phenotypic characterization of the SHR+BCAS+D-gal composite model. Specifically, we simultaneously evaluated spatial learning and memory, locomotor/exploratory activity, and motor-balance performance, and combined 7.0-T T2-weighted MRI, DTI-FA mapping, LFB staining, TEM analysis, corpus callosum MBP/NfL measurement, and serum NfL/NSE quantification. This design establishes a continuous evidence chain from behavioral abnormality to brain structural change, white matter microstructural injury, myelin ultrastructural disruption, axonal damage, and elevation of peripheral neural injury biomarkers. Therefore, the value of the present study is not merely that it repeats a three-factor modeling strategy, but that it systematically complements and expands the existing model by providing multidimensional validation of CSVD-related cognitive, behavioral, motor-balance, white matter, and biomarker phenotypes. Together, these complementary assessments extend the phenotypic characterization of the SHR+BCAS+D-gal model beyond that reported previously.

Behavioral phenotype is an important component for evaluating the clinical relevance of CSVD animal models. Previous studies have shown that CSVD is closely associated not only with vascular cognitive impairment but also with gait abnormalities, balance disorders, increased fall risk, and neuropsychiatric symptoms such as depression, apathy, and fatigue (Clancy et al., 2021; Su et al., 2022; Markus and de Leeuw, 2023). Accordingly, the present behavioral test battery was selected to assess complementary functional domains relevant to CSVD. The Morris water maze was used primarily to evaluate spatial learning and memory, the open field test was used to characterize spontaneous locomotor and exploratory activity together with central-zone behavior, and the balance beam test was used to assess motor coordination, balance, and task execution.

In the present study, the MWM results showed that escape latency was prolonged, platform crossings were reduced, and target-quadrant dwell time was shortened as hypertension, chronic cerebral hypoperfusion, and aging-related stress accumulated, indicating that composite risk factors significantly impair spatial learning and memory retention. Previous studies in chronic cerebral hypoperfusion models have also demonstrated that long-term reduction in cerebral blood flow can induce hippocampus-dependent learning and memory deficits, accompanied by white matter injury and glial activation. The open field test further showed decreased total distance traveled, central-zone activity, and rearing in model rats, suggesting impaired spontaneous locomotion and exploratory behavior, with possible anxiety-like or apathy-like tendencies. It should be noted that reduced central-zone activity may also be influenced by decreased overall locomotor ability; therefore, this result should be interpreted together with total distance traveled and exploratory indices. In the balance beam test, SHR+BCAS+D-gal rats showed prolonged crossing time, increased foot slips, and increased waiting and hesitation behaviors, suggesting that composite risk factors further impaired fine motor coordination and balance regulation. Collectively, these results indicate that the three-factor composite model reproduces the multidimensional neurological dysfunction commonly observed in CSVD patients, including cognitive decline, emotional/behavioral abnormality, and gait-balance impairment.

More generally, individual behavioral paradigms are not specific to a single neurological process. Morris water maze performance may be influenced by swimming ability, vision, motivation, and stress responses in addition to spatial memory. Similarly, open field and balance beam outcomes may reflect overlapping contributions from locomotor function, exploratory drive, motivation, and sensorimotor capacity. Alternative paradigms assessing similar domains, including the Y-maze or novel object recognition test for cognition, the elevated plus maze or light-dark box test for anxiety-related behavior, and the rotarod or quantitative gait analysis for motor coordination, might therefore yield partially different results. Accordingly, the behavioral findings of the present study should be interpreted together with the MRI, DTI, histopathological, and molecular findings rather than as isolated evidence of a specific behavioral construct.

The imaging phenotype further confirmed the detrimental effects of composite risk factors on brain structure and white matter microstructure. Structural MRI is an important tool for evaluating CSVD-related brain injury. In addition to white matter hyperintensities, lacunes, microbleeds, and enlarged perivascular spaces, brain atrophy is also recognized as an important imaging marker for CSVD burden assessment (Duering et al., 2023). Previous studies have shown that patients with CSVD may exhibit whole-brain or regional brain volume reduction, and that the degree of atrophy is closely related to cognitive decline and disease progression (Nitkunan et al., 2011; De Guio et al., 2020). In this study, T2-weighted MRI showed progressive enlargement of the lateral ventricles and reduction of hippocampal, cortical, and corpus callosum volumes with the accumulation of hypertension, chronic cerebral hypoperfusion, and aging-related stress. These findings suggest that the composite model can induce structural changes involving both gray matter regions related to cognition and major white matter fiber bundles. DTI provides a sensitive method for detecting microstructural white matter damage. Decreased FA generally reflects decreased fiber organization, demyelination, or axonal injury, and reduced FA has been closely associated with cognitive impairment, network disconnection, and disease burden in CSVD (Lawrence et al., 2014; Baykara et al., 2016). In the present study, FA values were reduced in the gcc, cingulum, dorsal hippocampus, and thalamic core region, with the most pronounced decline in the SHR+BCAS+D-gal group. These regions are closely related to interhemispheric communication, limbic-cognitive circuits, hippocampal memory function, and subcortical information integration. The reduction in FA therefore suggests that composite risk factors damage key white matter pathways and gray-white matter connectivity networks in CSVD-related cognitive and motor dysfunction.

Human CSVD is characterized by a heterogeneous spectrum of MRI-visible lesions, including white matter hyperintensities, lacunes, cerebral microbleeds, enlarged perivascular spaces, and brain atrophy, and clinical severity is often more closely related to the cumulative burden of these abnormalities than to any single lesion. In longitudinal human studies, the combined burden of white matter lesions, lacunes, and gray matter and hippocampal atrophy has been associated with subsequent cognitive and functional decline, while reduced white matter and hippocampal volumes have predicted incident dementia in patients with SVD (Jokinen et al., 2020; van Uden et al., 2016). Diffuse disruption of white matter integrity, including abnormalities involving the corpus callosum and other frontal-subcortical pathways, has also been associated with gait impairment and longitudinal gait decline (de Laat et al., 2011; van der Holst et al., 2018). In addition, circulating NfL levels have been related to active CSVD-associated brain injury, MRI lesion burden, and future dementia risk (Gattringer et al., 2017; Duering et al., 2018; Egle et al., 2021).

Against this clinical background, the hippocampal, cortical, and corpus callosum volume reductions, lower regional FA values, impaired spatial memory and balance-related performance, and elevated serum NfL observed in the present model parallel several structural, microstructural, functional, and biomarker features associated with increasing disease burden in patients with CSVD. Nevertheless, the present study was not designed to reproduce or quantify the complete spectrum of focal human CSVD lesions, particularly lacunes, cerebral microbleeds, enlarged perivascular spaces, or clinically defined white matter hyperintensities. The DTI and histopathological abnormalities observed in the rats should therefore be interpreted as evidence of diffuse white matter microstructural and myelin injury rather than as direct equivalents of every MRI-visible lesion found in human CSVD.

Histopathology and serum biomarkers further validated white matter and axonal injury induced by composite risk factors at morphological and molecular levels. White matter injury is one of the core pathological features of CSVD and is characterized by impaired myelin integrity, oligodendrocyte injury, axonal rarefaction, and disrupted fiber connectivity (Carriel et al., 2017). LFB staining is a classical histochemical method for evaluating myelin integrity, and reduced blue staining may reflect reduced myelin density or disrupted myelin organization. TEM permits qualitative visualization of ultrastructural abnormalities, including myelin lamellar loosening, irregularity, separation, and deformation (Chen et al., 2013). In the present study, the SHR+BCAS+D-gal group showed lighter LFB staining and more evident myelin lamellar loosening and deformation, consistent with the reductions in DTI-FA and MBP. MBP is a key structural protein required for myelin sheath stability, and decreased MBP is widely used as a marker of demyelination and white matter injury. In parallel, NfL is released into tissue fluid and peripheral blood after axonal cytoskeletal damage, and has been suggested as a biomarker related to CSVD burden and white matter injury (Duering et al., 2018; Peters, 2022). The present study found decreased MBP and increased NfL in the corpus callosum, as well as elevated serum NfL and NSE, in the composite model. These findings indicate that the model not only reproduces myelin injury but also reflects axonal and neuronal injury-related molecular changes, thereby improving the translational relevance of the model for CSVD-related neural damage.

In addition to white matter and axonal injury, this study further showed that composite risk factors may induce BBB-related structural protein abnormalities, neuroinflammatory activation, and oxidative stress imbalance in the hippocampus and frontal cortex. BBB dysfunction is considered a key event in CSVD pathogenesis, as it can lead to plasma component extravasation, disruption of neurovascular unit homeostasis, and subsequent white matter and neuronal injury (Rajeev et al., 2022; Jia et al., 2024). Notably, studies using *Atp11b*-deficient rat models have demonstrated molecular and morphological abnormalities in cerebral endothelial cells together with white matter vulnerability, even in the absence of hypertension, supporting the possibility that endothelial dysfunction represents an early component of SVD pathogenesis (Rajani et al., 2018; Quick et al., 2022). Claudin-5 and ZO-1 are important tight-junction proteins that maintain BBB integrity. Reduced expression of these proteins suggests impaired tight-junction structure and increased BBB vulnerability. In this study, Claudin-5 and ZO-1 levels decreased progressively in the hippocampus and frontal cortex after the introduction of BCAS and D-gal, suggesting that the composite model may aggravate BBB-related pathological changes on a hypertensive background.

After BBB damage, the brain parenchymal microenvironment becomes more susceptible to circulating inflammatory mediators, oxidative metabolites, and extravasated plasma proteins, thereby triggering glial responses and neuroinflammatory cascades. Iba1 and GFAP are commonly used to reflect the reactive states of microglia and astrocytes, respectively. Previous studies of chronic cerebral hypoperfusion have also suggested that abnormal interactions among neurons, astrocytes, and microglia may contribute to ischemia-related neurodegenerative injury (Lana et al., 2014). The present study observed elevated Iba1 and GFAP levels in the hippocampus and frontal cortex, suggesting glial activation in the composite model. Meanwhile, increased MDA and decreased SOD indicated oxidative stress imbalance. Oxidative stress can directly damage lipids, proteins, and nucleic acids, and can amplify BBB disruption, neuroinflammation, and myelin injury (Kimura et al., 2025). Therefore, the pathological phenotype of the SHR+BCAS+D-gal model may involve the interaction of vascular injury, BBB disruption, glial inflammation, oxidative stress, and white matter damage, forming a continuous pathological cascade from vascular risk factors to neural network dysfunction.

Several limitations should be considered. First, the present study included only male rats. This choice was made prospectively for an initial proof-of-concept and multimodal characterization of the composite model within a defined SHR/WKY background, in which the BCAS procedure and perioperative parameters were optimized for male rats within a relatively narrow body-weight range. Nevertheless, this design represents an important scientific limitation rather than merely a practical constraint. The absence of female animals means that sex was not incorporated as a biological variable, and the present findings should not be assumed to establish sex-general validity of the model. This limitation is particularly relevant because sex-related differences have been reported in CSVD severity, risk-factor associations, and clinical outcomes, and sex-inclusive design and sex-disaggregated reporting are increasingly emphasized in preclinical research (Jiménez-Sánchez et al., 2021; Finn et al., 2026). We also recognize that using males only may contribute to a male-as-default framework if not explicitly acknowledged. Future studies should therefore include both male and female animals in a prospectively balanced design to determine whether sex influences model induction, mortality, phenotypic severity, neuroimaging abnormalities, histopathological injury, and molecular responses. Second, composite models may inherently exhibit greater biological and experimental heterogeneity than single-insult models. In the present study, individual variability in the hypertensive background, the severity of BCAS-induced stenosis and hypoperfusion, and the response to D-gal may have accumulated and contributed to increased within-group variability. Moreover, because the current sequential grouping design was not a complete factorial design, the independent, additive, and interactive contributions of hypertension, chronic cerebral hypoperfusion, and aging-related stress could not be distinguished. Therefore, although this composite model is useful for characterizing CSVD-like phenotypes under a multifactorial context, single-insult or factorially designed models may be more appropriate for isolating specific disease hallmarks and identifying the mechanisms independently driven by individual risk factors. Third, although the selected behavioral paradigms provided complementary assessments of spatial learning and memory, locomotor/exploratory activity, and motor-balance performance, none of these tests is specific to a single neurological construct. Performance may be influenced by locomotor capacity, motivation, stress responses, sensory function, and task demands. In addition, the present behavioral battery did not include a dedicated assessment of recognition memory or episodic-like memory. Tests such as the novel object recognition test or related object-location/object-place paradigms would provide additional information on cognitive domains not fully captured by the Morris water maze. Furthermore, behavioral assessments were performed only once after completion of model establishment. Although this endpoint design allowed the behavioral findings to be aligned with MRI, histopathological, and molecular assessments, it did not capture the temporal evolution of cognitive, exploratory, or motor-balance abnormalities during model development. Repeated longitudinal behavioral testing could help clarify the onset, progression, and stabilization of functional impairment in relation to structural and molecular pathological changes. However, repeated exposure to behavioral paradigms may also introduce learning, habituation, stress, or training effects. Future studies should therefore incorporate carefully designed longitudinal behavioral assessments together with serial MRI and biomarker measurements to better characterize the dynamic progression of this composite model. Accordingly, the present behavioral findings should be interpreted in conjunction with the imaging, histopathological, and molecular evidence. Fourth, although T2-weighted MRI, DTI, LFB staining, and TEM were used to characterize brain structure and white matter injury, cerebral perfusion imaging, vascular reactivity testing, and longitudinal dynamic MRI were not included. The temporal relationship between cerebral blood flow changes and structural injury during model formation therefore requires further clarification. Fifth, although ELISA was used to detect representative markers related to BBB integrity, neuroinflammation, oxidative stress, myelin injury, axonal injury, and neuronal damage, this study did not further investigate upstream and downstream signaling pathways or cell-type-specific mechanisms. Future studies should combine immunofluorescence, transcriptomics, proteomics, and cell-specific labeling to clarify the dynamic interactions among endothelial cells, pericytes, astrocytes, microglia, oligodendrocytes, and neurons in this model. Finally, although the SHR+BCAS+D-gal model is closer to the multifactorial clinical context of CSVD, no animal model can fully reproduce the heterogeneity of human CSVD. Therefore, interpretation and application of this model should be integrated with the specific scientific question being addressed. In addition, TEM was used for qualitative ultrastructural assessment, and quantitative morphometric indices, such as the g-ratio or the proportion of abnormal myelinated fibers, were not available. Future studies should incorporate systematic field sampling and blinded quantitative morphometry to provide a more objective assessment of myelin injury.

In conclusion, this study established and characterized an SHR+BCAS+D-gal three-factor composite CSVD model in male rats by integrating hypertension, chronic cerebral hypoperfusion, and aging-related stress. The model showed progressive cognitive decline, reduced exploratory behavior, and motor-balance impairment, together with structural changes in core brain regions, reduced white matter fiber integrity, myelin and axonal injury, abnormal BBB-related protein expression, glial activation, and oxidative stress imbalance. These findings suggest that, in male rats, the SHR+BCAS+D-gal composite model recapitulates several key behavioral, neuroimaging, histopathological, and molecular features relevant to clinical CSVD. Thus, within this defined male experimental background, the model may provide a useful platform for studying mechanisms underlying the chronic progression of CSVD and for evaluating potential therapeutic strategies targeting BBB protection, neuroinflammatory regulation, oxidative stress reduction, and white matter repair. However, the present findings are restricted to male animals, and prospective validation in female rats is required before the model can be considered generalizable across sexes.

## Data Availability

The original contributions presented in this study are included in the article/Supplementary material, further inquiries can be directed to the corresponding authors.
